# Examining the effects of climate and environmental hazards on vector and water-borne diseases in Eastern Uganda

**DOI:** 10.1007/s44274-026-00616-4

**Published:** 2026-03-07

**Authors:** Revocatus Twinomuhangi, Hakimu Sseviiri, Susannah H. Mayhew, PARES Research Group, Mateus Kambale Sahani

**Affiliations:** 1 Department of Geography, Geo-Informatics, and Climatic Sciences, School of Forestry, Environmental and Geographical Sciences, College of Environmental and Agricultural Sciences, Makerere University, Kampala, Uganda; 2 Department of Global Health and Development, Faculty of Public Health and Policy, London School of Hygiene & Tropical Medicine (LSHTM), London, UK

**Keywords:** Environmental hazards, Public health, Crisis responses, Infectious diseases, Floods, Mitigation measures, Medical interventions, Uganda

## Abstract

**Background:**

Environmental hazards like floods, droughts and landslides pose serious public health and wellbeing consequences for populations, especially vulnerable communities in countries with low adaptive capacity. Empirical evidence from peer-reviewed literature and official climate change assessments indicates that, alongside high interannual climate variability, Uganda has experienced observable climate change over the past 50 years. This changing and increasingly variable climate poses challenges to population health in diverse ways, including through the transmission of infectious diseases. This study explored local perceptions of the impact of climate variability and change on the prevalence of infectious diseases like typhoid fever, malaria, and diarrhoeal diseases in three districts: Amudat, Bududa, and Katakwi in Eastern Uganda. Relatively few integrated and empirical studies have been conducted in Uganda to assess the specific climate-related health risks faced by local communities.

**Methods:**

A cross-sectional survey of 341 respondents was conducted to collect data from households. Data were collected using Kobo software, exported, cleaned and analysed with SPSS 28.0

**Results:**

Populations in the three study districts perceived drought, floods, and landslides as significant hazards, though with varying levels of exposure, frequency, and severity. Strong associations were found between environmental hazards and occurrences of infectious diseases. Floods, significantly increased the reported prevalence of malaria (Chi-Square = 12.901, p < 0.005), typhoid fever (Chi-Square = 14.215, p < 0.003), and diarrhoeal diseases (Chi-Square = 8.407, p < 0.038). Similarly, drought severity is significantly associated with higher rates of diarrhoeal diseases (Chi-Square = 14.548, p < 0.002), whereas no significant associations were observed for malaria (Chi-Square = 4.182, p > 0.242) or typhoid fever (Chi-Square = 4.739, p > 0.192). Landslides were significantly associated with diarrheal diseases (Chi-Square = 7.846, p < 0.049), but not with malaria (Chi-Square = 2.603, p > 0.457) or typhoid fever (Chi-Square = 3.277, p > 0.351). Most respondents experienced the negative interactive effects of multiple environmental hazards on their health status. The increased prevalence of diarrhea was attributable to floods, drought, and landslides; cases of typhoid fever were increased by floods rather than by drought or landslides. Malaria was more influenced by floods.

**Conclusion:**

Environmental hazards affected population health in the three districts by increasing the risk of waterborne diseases, such as diarrhea, during severe droughts and landslides. Floods exacerbated waterborne and vector-borne diseases by creating ideal conditions for mosquito breeding and water contamination. There is an urgent need for multihazard-targeted interventions, including improved access to water, sanitation, and hygiene (e.g., regular handwashing with soap, safe water access and use, boiling water, proper sanitation, community WASH sensitization supported by local public health outreach), as well as disaster preparedness strategies, to reduce the health burdens of environmental hazards and enhance community resilience in these regions.

## Introduction

Climate change is a serious threat worldwide impacting the population health by increasing the frequency of vector-borne and zoonotic diseases [1–3]. It is a serious issue affecting Sub-Saharan African countries with immediate consequences on health and wellbeing of the population, and more especially because these countries have low adaptive capacity [4–8]. The effects of climate change and extreme weather events such as more intense and frequent droughts, rising temperatures, heat waves, rainstorms, flooding and landslides have been reported with flooding being a severe problem in countries such as Madagascar, Mozambique, and Uganda, leading to internally displaced populations, homeless people, injury, diseases and even deaths [7, 9–11]. 

Climate change is occurring in Uganda, and Eastern Uganda is no exception. Over the past century, Uganda has experienced statistically significant warming, rising potential evapotranspiration and atmospheric humidity, more frequent rainfall extremes (intense downpours and longer dry spells), and detectable shifts in wind patterns and cloud cover, changes that exceed natural variability and are consistent with an anthropogenic climate-change signal [12–14]. For example, between 1900 and 2009, temperatures increased by 0.8 to 1.3 °C, and annual rainfall reduced at an average rate of 3.5% per decade since 1960. While Uganda continues to experience high interannual climate variability, the persistent upward trends in temperature, evapotranspiration, humidity, and rainfall extremes over the past three decades indicate a longer-term climate-change signal that cannot be explained by variability alone. Temperatures are projected to increase by 1.8 °C by the 2050s and by 3.7 °C by the 2090s [12–14]. Further, Uganda is among the world’s most vulnerable countries to climate change impacts and is ranked 36th most vulnerable and 163rd most ready country to adapt [15–17]. Eastern Uganda is highly vulnerable to floods, landslides and droughts [18]. Floods are most common, with severe flooding episodes associated with above-average rainfall in 2007, 2010, 2019, and during the 2020–2021 La Niña period, more especially in low-lying districts in the Teso subregion, causing displacement of people, crop destruction, waterborne diseases, and infrastructure damage [19, 20]. While seasonal flooding in the region is a long-standing feature linked to bimodal rainfall patterns and climate variability, such as El Niño–Southern Oscillation (ENSO), several studies suggest that recent events are occurring more frequently and with greater intensity [21]. Over the past two decades, droughts have been more severe and frequent in Eastern Uganda, with notable events that include the prolonged drought conditions during 2016–2017 and 2020–2022, and in the Teso and Karamoja sub-regions, they have led to water scarcity, crop failures, and livestock losses [19, 20]. Landslides on the other hand are more frequent in the Mt. Elgon region, largely driven by intense rainfall, deforestation, and steep slopes [22].

Evidence exists that links the effects of climate change to ill-health in Uganda [23]. For example, rising temperatures, are creating favorable conditions for the breeding and survival of mosquitos that spread malaria, even in high altitude areas of South-Western Uganda that were previously malaria-free due to their cold temperatures [20, 24]. There is an increase in cases of malaria that may be linked to climate hazards in Bududa, an area that was not previously exposed to the disease and its population not aware of community based malaria prevention measures [19]. Further, there are reported increase of malaria cases up to 30% in the immediate post-crisis period of severe flooding event in Western Uganda [20]. The upsurge in malarial cases across the country has led the disease to be more epidemic (sporadic) in nature, increasing the costs of its management from between USD 0.7-15.8 million in 2010 to between USD 1.55 and 41.7million in 2050 [25].

Cholera outbreaks are becoming increasingly prevalent as abnormal rainfall patterns lead to drought, and then intense rainfall and flooding which displaces populations and overwhelms environmental sanitation capabilities [26]. A study conducted in Kampala found that drinking water from unprotected water sources led to cholera epidemic which was socially constructed due to risky behaviour of people discharging faecal matter from their on-site sanitation facilities into drainage connected to well/s [27]. Furthermore, Bududa District experienced cholera epidemic in 2019 that occurred two weeks after flooding event due to heavy rain and resulted in one death even though it was controlled by the response team from the Ministry of Health-Uganda [28]. Water, Sanitation, and Hygiene (WASH) facilities in Bududa District were in a chaotic state, and the disaster affected 22 parishes [28]. Besides, communication for floods preparedness was not completely used by local people in Katakwi District due to partial delivery and use of technical language that hindered local comprehension [29]. Repeated outbreaks of cholera were reported in Bududa District due to lack of access to protected water sources. People were using water from River Manafwa without boiling it because it was a social habit and therefore, they did not find it useful to boil water [30]. This exposed them to diverse enteric infections [30–32]. The burden of typhoid fever is also rising due to contaminated water sources that are mainly associated with flooding occurrences [26].

With the projected future change in climate, the burdens of diseases is expected to worsen from the current state especially for malaria (37%), intestinal worms (5.4%), skin diseases (3.2%), cholera (3.13%), and acute diarrhea (3%) [33, 34]. There are relatively few integrated, empirical studies conducted in Uganda (e.g. see ref [26, 28–30]), especially in districts that are highly vulnerable to one or more climatic hazards, that generate quantitative evidence on the association between climate variability and change and infectious diseases. This study contributes to filling this knowledge gap by exploring the perceived effect of climate and environmental hazards on the health status of the population, especially on infectious diseases, i.e., malaria, diarrhoeal diseases, and typhoid fever, in three districts of Uganda, which were particularly prone to drought, landslides, and flooding. The targeted districts were Amudat, Bududa, and Katakwi in Eastern Uganda, and their choice was informed by recurrent exposure to multiple and interconnected climatic hazards that, in most cases, resulted into health burdens to the populations. We therefore sought to respond to three research questions. First, to what extent are people in the three districts exposed to different environmental hazards? Second, does the exposure to environmental hazards lead to reported increases in infectious diseases? Third, how does the severity of environmental hazards affect the frequency of reported infectious diseases?

Besides bridging the knowledge gap on climate and environment-related disease risks in Eastern Uganda, the study makes a theoretical contribution to the climate–health literature in two ways. First, it expands climate-health pathways theory by showing that climate and environmental hazards, such as drought, flooding, and landslides, are not merely background conditions but independent and interacting drivers of vector and waterborne diseases. Second, the study advances vulnerability theory by indicating that climate and environmental hazards exert strong effects on disease outcomes independent of key social and demographic characteristics, reinforcing the primacy of place-based ecological conditions over individual factors in shaping infectious disease risk. By making these contributions, the study provides an explanation of how climate and environmental factors affect disease transmission in regions that are highly sensitive to climate and environmental change.

## Methods

### Study location and population

**Fig. 1:**
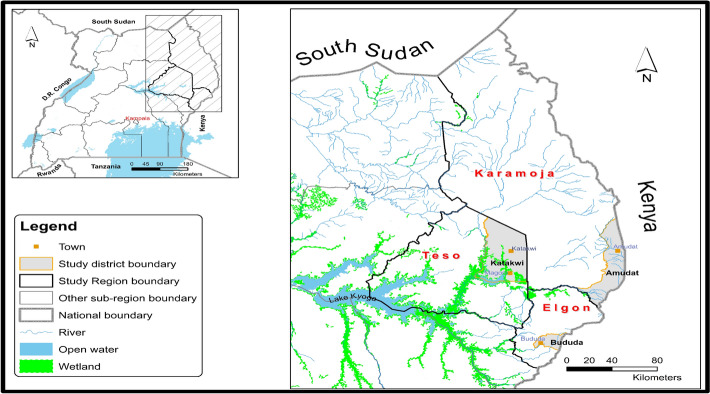
Map showing study regions and districts [Generated by authors using geospatial data from Uganda National Bureau of Statistics (UBOS)]

This study was conducted in three districts from three sub-regions in Eastern Uganda which are Amudat from Karamoja sub-region, Bududa from Mt Elgon sub-region, and Katakwi from Teso sub-region. These regions were chosen for their particularity and exposure to recurrent, frequent, and severe climate change and environment related hazards, shocks and stresses for a long period [18]. Karamoja sub-region is semi-arid and is associated with environmental and climate extremes such as desertification, ecosystem degradation, high temperatures, flash floods, recurrent droughts, and unreliable rainfall. Teso sub-region experiences frequent severe drought and flooding that are directly linked to famine, displacement, and loss of lives. The Mt. Elgon region is largely mountainous and densely populated where intense rainfall, flooding, and landslides are the common environmental challenges. These three sub- regions are shown in Fig. 1 below.

### Study design and sample size

Data were collected through a cross-sectional survey database of 379 household participants (1 participant was selected per household) from a completed project implemented by Makerere University Centre for Climate Change Research and Innovations (MUCCRI) and the International Organisation of Migration (IOM) in collaboration with the Ministry of Water and Environment (MWE) and the Uganda Bureau of Statistics (UBOS). From the survey database of 379 participants, 341 respondents were included in the analysis from the 3 selected districts (Amudat, Bududa, and Katakwi). Participants were included due to the completeness of data according to the study dataset variables.

The total parish household population of 30,650 people of all spaces of vulnerability (Table 1) that were identified during a stakeholder consultative process was used to calculate the study sample size using the Krejcie and Mogan [35] formula/tables to generate the sample size. In Uganda, Parishes are administrative subdivisions positioned between sub-counties and villages within the nation's decentralized local government framework. The parish population was prioritized since it is the lowest administrative unit that is factored into district, regional and national development planning, and service delivery to the populations. The equation used to generate the sample size is expressed as follows:

$$S=\frac{{\mathrm{X}}^{2}Np\left(1-p\right)}{{\mathrm{d}}^{2}(N-1)} +{\mathrm{X}}^{2}p(1-p)$$

where;

**Table 1:** Population characteristics of the most vulnerable areas/spaces identified during stakeholder consultations

Region	District	Sub-county	Parish	Population	Total study population
Karamoja	Amudat	Karita	Lokales	11,360	18,029
		Loroo	Achorichori	6669	
Elgon	Katakwi	Toroma	Akurao	2419	7298
		Magoro	Omasia	4879	
Mt. Elgon	Bududa	Bundensi	Nametsi	726	5323
		Bushika	Bufutsa	4597	
Total				30,650	30,650

Source: Uganda Bureau of Statistics (https://www.ubos.org/explore-statistics/20/)

S = required sample size; *X*^*2*^ = the table value of Chi-square for 1 degree of freedom at the desired confidence level of 0.05 = 3.841; N = the population size (30,650); p = the population proportion (assumed to be 0.50) since this would provide the maximum sample size; d= the degree of accuracy expressed as proportion (0.05).

While the sample size considered after calculation was 379 participants, 38 participants could not be included in the analysis due to lack of some data (missing data). As a result, a total of 341 participants were included in the analysis. Despite such a shortfall, we were able to reach around 90% of our initial planned study sample and the data could be reliably used to derive analyses, interpretations, and conclusions.

### Data collection, management, analysis, and presentation

For this study, we used an existing dataset that was created with survey data collected by Makerere University supported by IOM and Uganda Ministry of Environment to inform their project planning. We developed a new dataset for this paper that was created in SPSS 28.0 for data cleaning and analysis, the initial one serving as the data source. Data selection and entry to the new database were performed from 10 August 2023 to 31 March 2024. Data were collected for demographic variables such as age group, gender (sex), educational level, marital status. In addition, variables related to severity of drought, flood, and landslides were also included in the new database. Furthermore, outcome variables related to cases of malaria, diarrhea, and typhoid fever (coded according to reported presence of the disease: Yes/No) were incorporated in the dataset of this study. The data analysis was performed using SPSS software v28.0 which was both univariate analysis (proportions were used) and bivariate (means used). Univariate analysis was performed for independent variables which are the ones related directly to environment hazards and the dependent variable which are the ones related to the three diseases. Bivariate analysis was conducted to examine the link between dependent and independent variables. For univariate analysis, One-Sample proportion test was performed to determine the significance between the observed rates of the three diseases and the national rates of each disease and One-Sample Chi-square test to determine the significance between the different categories within the same variable for floods, landslide, and drought while the main Chi-Square test was performed for bivariate analysis. It is documented that expected national rate of malaria in Uganda is 267.4 per 1000 people (26.74%) [36, 37], 160 cases per 100,000 people (0.16%) for typhoid fever [38, 39], and 18.6% for diarrhoeal diseases in general (much higher for children under-5, 22%) [37, 40].

The findings of the study are presented in bar charts for univariate analysis and in tables for bivariate analysis.

### Study limitations

The results presented in this study may not show the current prevalence of these diseases as respondents gave their experience for a long period covering a full decade. Hence, inference analysis is not possible for this manuscript. This is a limitation of our study, and we would need another study collecting clinical data in hospitals and comparing these data between the rainy and dry seasons. The findings from this study open avenues for further investigation to address this gap.

## Results

### Socio-demographic characteristics

Table 2 below summarises the demographic characteristics of participants to this study.

**Table 2:** Demographic characteristics of study participants

Characteristics	Description	Frequency (n)	Percentage (%)	Characteristics	Description	Frequency (n)	Percentage (%)
Gender	Male	206	60	Education level	No formal education	231	68
	Female	135	40		Completed primary education	72	21
					Completed secondary education	23	7
Age groups (years)	18–25	35	10		Obtained tertiary education (diploma)	10	3
	26–35	85	25		Advanced Secondary education	5	2
	36–45	64	19				
	46–60	91	26	Residential address	Katakwi district	132	39
	> 60	66	19		Bududa district	130	38
					Amudat district	79	23
Marital status	Married	270	79	Duration of stay in the region	0–9 years	4	1.2
	Widowed	34	10		10–20 years	21	6.2
	Divorced/separated	19	6		21–40 years	135	39.6
	Unmarried	18	5		41–60 years	128	37.5
					60 years and more	53	15.5

In general, the minimum age of the participants was 18 years old and maximum age was 90 years old. The median age was 45 years old while the standard deviation was 16.319, and the mean of age was 45.34. Regarding the duration of stay in the study area, the mean was 42.72 and the standard deviation 16.732. The minimum duration was 3 years, the median duration 41, and the maximum duration of 85 years. The survey was mostly attended by young people and early adults who spent 21 to 40 years in the area (42.6%) followed by those who spent 41 to 60 years (33.9%).

### Environmental hazards exposure

**Fig. 2:**
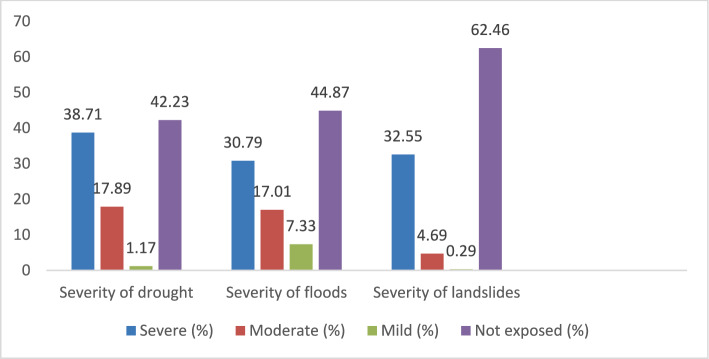
Percentage of people who experienced severity of drought, floods, and landslide, by reported severity in the three districts

Respondents reported that they were exposed to several environment-related hazards including drought, floods, and landslides as the predominant environmental stressors experienced during the last decade, with varying degree of severity as indicated in Fig. 2 below.

The study examined self-reported perceptions of whether respondents were affected by environmental hazards. At least 57.77% (n = 197) of respondents reported being affected by drought in the three districts while 42.23% (n = 144) were not. Of those indicating that they had been affected, two-thirds (n = 132) of people were severely affected and one-third of them were both moderately (n = 61) and mildly (n = 4) affected. There is significant difference between these observations as determined by One-Sample Chi-Square test (p < 0.001).

At least 55.13% (n = 188) of respondents were affected (n = 105 severely affected; n = 58 moderately affected; n = 25 mildly affected) by floods while 44.87% (n = 153) were not affected. There was a significant difference between these categories (severe, moderate, and mild) as determined by One-Sample Chi-Square test (p < 0.001). This means that most respondents felt the effects of floods.

37.54% (n = 128) of respondents reported being affected by severe landslides while 62.46% (n = 213) said they were not. Among those who reported being affected (n = 111 severely affected; n = 16 moderately affected; n = 1 mildly affected). There is significant difference between these three categories as determined by One-Sample Chi-Square test (p < 0.001). This means that most respondents felt the effects of landslides (more people severely affected than other categories).

### Infectious diseases compared to national rates

We explored the reported experience of respondents in terms of three diseases (malaria, diarrhea, and typhoid fever) and their association with climate-related hazards in their area, compared to the known national situation of the three diseases.

**Fig. 3:**
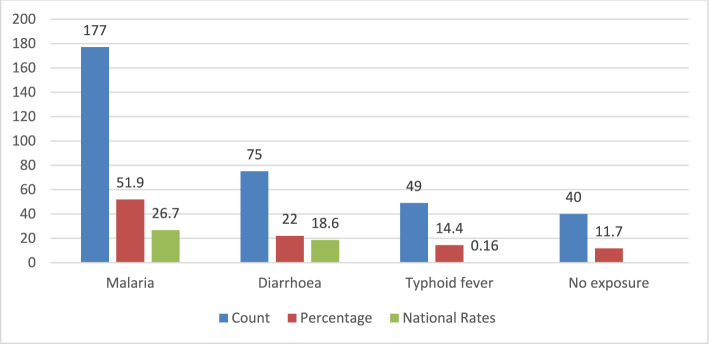
Reported rates of malaria, diarrhea, and typhoid fever after exposure to climate/environmental hazards versus the national rates [N (sample size) = 341]

Climate hazards such as floods, drought, and landslide are associated with the population’s health by increasing cases of the three infectious diseases of epidemiological characteristics stated above (self-reported) in the three districts of the study compared to the known national rates. These associations are highlighted in Fig. 3 below. The respondents were asked to specify which diseases they experienced following a climate hazard. Whereas three focal diseases, i.e., diarrhoea, typhoid fever, and malaria (the three diseases in this study), were mentioned, respondents also mentioned some other diseases, including foot diseases, lung diseases, ticks, skin diseases, and mental stress. Although these other diseases were documented, they were excluded from the primary analysis because they did not align with the study’s predefined objectives.

A higher proportion of malaria cases was observed in the region than the expected national rate, with a 1.9-fold increase. The difference between the observed rate and the expected national rate was statistically significant as shown by the One-Sample Mid-p adjusted Binomial Test (95%CI [0.446–0.52], p < 0.001). In addition, the proportion of diarrheal diseases was higher than the national rate by 1.2-fold with a statistically significant difference, 95%CI [0.179–0.267], p < 0.001. Furthermore, typhoid fever situation was worse than the national situation, with a rate 90-fold higher than expected (95% CI [0.110–0.185], p = 0.016).

### Reported effect of environmental hazard severity on infectious diseases

As epidemic diseases (malaria, diarrhea, and typhoid fever) were reported after climate events, we wanted to explore the association of these diseases with the reported severity of the climate hazard.

#### Severity of floods and infectious diseases

We explored the effect of floods on the reported occurrence of the three diseases. Findings are highlighted below in Table 3. The results demonstrate that the reported number of malaria cases increases with the reported severity of floods. There is a higher rate of malaria in people who were exposed to very severe floods, it becomes moderate in those who were exposed to severe floods, and lower in those who were exposed to not severe floods. The difference observed is statistically significant, Chi-Square = 12.901, p = 0.005. The same situation has been observed for cholera or diarrheal diseases, and for typhoid fever (Chi-Square = 8.407, p = 0.038 and Chi-Square (Fisher’ Exact Test) = 13.254, p = 0.003 respectively).

**Table 3:** Reported Severity of floods and infectious diseases

				Severity of floods			Chi-Square & p value
		Severe	Moderate	Mild	No exposure	Total	
Malaria	No	36 (22.0%)	35 (21.3%)	14 (8.5%)	79 (48.2%)	164 (100%)	Chi-Square (Pearson) = 12.901, p = 0.005*
	Yes	69 (39.0%)	23 (13.0%)	11 (6.2%)	74 (41.8%)	177 (100%)	
	Total	105 (30.8%)	58 (17.0%)	25 (7.3%)	153 (44.9)	341 (100%)	
Diarrheal diseases	No	76 (28.6%)	52 (19.5%)	22 (8.3%)	116 (43.6%)	266 (100%)	Chi-Square (Pearson) = 8.407, p = 0.038*
	Yes	29 (38.7%)	6 (8.0%)	3 (4.0%)	37 (49.3%)	75 (100%)	
	Total	105 (30.8%)	58 (17.0%)	25 (7.3%)	153 (44.9)	341 (100%)	
Typhoid fever	No	79 (27.1%)	53 (18.2%)	21 (7.2%)	139 (47.6%)	292 (100%)	Chi-Square (F. Exact) = 13.254, p = 0.003*
	Yes	26 (53.1%)	5 (10.2%)	4 (8.2%)	14 (28.6%)	49 (100%)	
	Total	105 (30.8%)	58 (17.0%)	25 (7.3%)	153 (44.9)	341 (100%)	

Note: For diarrhea, the assumption for Chi-Square Pearson test was met even though one cell has a value less than 5. This is because the expected count was 5.50 while running the test

#### Severity of landslides and infectious diseases

The study explored the reported effect of landslides on the occurrence of the 3 infectious diseases. The findings of our investigations are presented below in Table 4. The results reveal a significant association between reported landslide severity and diarrheal diseases while no significant relationships were observed for malaria and typhoid fever. The reported number of malaria cases were not influenced by the severity of landslides. The rate of malaria in people who were exposed to very severe landslides were almost the same as for those who were less exposed or not exposed. The difference between these observations was not statistically significant, Chi-Square (Fisher’s Exact Test) = 2.517, p = 0.461. The same situation was observed for reported typhoid fever (Chi-Square [Fisher’s Exact Test] = 3.238, p = 0.392). However, the severity of landslides had an effect on the cases of reported diarrheal diseases with an increase in cases for people who were more exposed than those who were less or not exposed. There is a statistically significant difference between these observations (Chi-Square = 7.939; p = 0.034). This means people who reported being severely exposed to severe landslides also reported a higher prevalence of diarrhea than those who were less exposed. This may be explained by contamination of water sources during landslides and highlight the need to prioritize interventions addressing diarrheal diseases in areas prone to severe landslides.

**Table 4:** Reported severity of landslides and infectious diseases

				Severity of landslides			Chi-Square & p value
		Severe	Moderate	Mild	No exposure	Total	
Malaria	No	50 (30.5%)	6 (3.7%)	1 (0.6%)	107 (65.2%)	164 (100%)	Chi-Square (F. Exact) = 2.517, p = 0.461
	Yes	61 (34.5%)	10 (5.6%)	0 (0.0%)	106 (59.9%)	177 (100%)	
	Total	111 (32.6%)	16 (17.0%)	1 (0.3%)	213 (44.9)	341 (100%)	
Diarrheal diseases	No	77 (28.9%)	12 (4.5%)	1 (0.4%)	176 (66.2%)	266 (100%)	Chi-Square (F. Exact) = 7.939, p = 0.034
	Yes	34 (45.3%)	4 (5.3%)	0 (0.0%)	37 (49.3%)	75 (100%)	
	Total	111 (32.6%)	16 (4.7%)	1 (0.3%)	213 (62.5)	341 (100%)	
Typhoid fever	No	99 (33.9%)	15 (5.1%)	1 (0.3%)	177 (60.6%)	292 (100%)	Chi-Square (F. Exact) = 3.238, p = 0.392
	Yes	12 (24.5%)	1 (2.0%)	0 (0.0%)	36 (73.5%)	49 (100%)	
	Total	111 (32.6%)	16 (4.7%)	1 (0.3%)	213 (62.5%)	341 (100%)	

#### Severity of drought and infectious diseases

The study investigated the reported effect of severe drought of the occurrence of the three infectious diseases. Table 5 summarises the reported effect of severe drought on malaria, diarrhea and typhoid fever. This table showed that the reported number of malaria cases were not influenced by the reported severity of drought. The reported rate of malaria in people who said they were exposed to very severe drought being almost the same as for those who said they were less exposed or not exposed. The difference was not statistically significant, (Chi-Square = 4.104, p = 0.259). The same situation was observed for typhoid fever (Chi-Square = 4.312, p = 0.208). However, the reported severity of drought showed a negative effect on the reported cases of diarrheal diseases with an increase of cases in people who said they were more exposed than those who said they were less or not exposed. There is a statistically significant difference between these observations (Chi-Square = 15.616, p < 0.001). This means people who reported being severely exposed to drought had a higher prevalence of diarrheal diseases than those who reported being less exposed.

**Table 5:** Reported severity of drought and infectious diseases

				Severity of drought			Chi-Square & p value
		Severe	Moderate	Mild	No exposure	Total	
Malaria	No	72 (43.9%)	27 (16.5%)	1 (0.6%)	64 (39.0%)	164 (100%)	Chi-Square (F. Exact) = 4.104, p = 0.259
	Yes	60 (33.9%)	34 (19.2%)	3 (1.7%)	80 (45.2%)	177 (100%)	
	Total	132 (38.7%)	61 (17.9%)	4 (1.2%)	144 (42.2)	341 (100%)	
Diarrheal diseases	No	177 (44.0%)	45 (16.9%)	3 (1.1%)	101 (38.0%)	266 (100%)	Chi-Square (F. Exact) = 15.616, p < 0.001
	Yes	15 (20.0%)	16 (21.3%)	1 (1.3%)	43 (57.3%)	75 (100%)	
	Total	132 (38.7%)	61 (17.9%)	4 (1.2%)	144 (42.2%)	341 (100%)	
Typhoid fever	No	113 (38.7%)	51 (17.5%)	2 (0.7%)	126 (43.2%)	292 (100%)	Chi-Square (F. Exact) = 4.312, p = 0.208
	Yes	19 (38.8%)	10 (20.4%)	2 (4.1%)	18 (36.7%)	49 (100%)	
	Total	132 (38.7%)	61 (17.9%)	4 (1.2%)	144 (42.2%)	341 (100%)	

## Discussion

The study showed respondents' experiences with diverse environmental hazards, including droughts, floods, and landslides. The populations across (between and among) the study areas reported experiences of severe drought, floods, and landslides and reported associated vector-borne and water-borne disease occurrence in Eastern Uganda, albeit with varying levels of exposure and severity. These observations highlight the interconnectedness of climate and environmental hazards, as well as the disproportionate health burden they place on vulnerable populations. The results reflect perceived linkages rather than causal relationships and highlight how households relate disease occurrence to changes in local climatic and environmental conditions. Such findings confirm existing literature on complex disasters where for example floods and drought events in the Horn of Africa have been found not to occur or act in isolation and yet fragile contextual conditions tend to further aggravate their impacts [32]. Other studies also document multiple climatic/environmental hazards facing vulnerable populations and pose serious health challenges [31, 41–43].

The study found that reports of diarrheal diseases were associated with reported severity of landslides, with individuals exposed to severe landslide reporting more cases compared to those less exposed. The significant association between landslides and diarrheal diseases can be explained by the contamination of water sources during landslides. This may be explained by lack of reliable sources of water, people using water from the river, sharing of water sources with animals/livestock and dependence on temporarily improvised water sources dug at riverbeds during the dry season. Landslide often disrupts infrastructure, including sanitation systems, and lead to the washing of debris and pathogens into water sources/supplies, making communities more susceptible to waterborne diseases.

The absence of significant reported association for malaria and typhoid fever suggests that landslides do not directly affect the environmental conditions required for their transmission, such as stagnant water for mosquito breeding or long-term contamination of water supplies/sources. These findings align with existing literature which has noted that landslides frequently disrupt water and sanitation systems, particularly in resource-limited settings, increasing the risk of diarrheal diseases [44–47]. Similarly, studies have emphasized that extreme weather events, including landslides, exacerbate the spread of waterborne diseases due to compromised water quality and poor hygiene practices [48–51]. The lack of significance for people who were exposed to landslides and who also reported becoming sick of malaria aligns with evidence that malaria transmission is more strongly influenced by stagnant water and vector habitats than by geological events such as landslides [52]. It has been found that geophysical topographic indicators account for 67% of vector density variance but with a below average infection prevalence (43%) observed [52]. It is highly probable that although highlands might provide a conducive environment for mosquito breeding, the conditions might lower infection rates by narrowing the lifecycle of mosquitoes and yet the prevalence of dispersed settlements inhibits accelerated transmission rates. The findings underscore the need to prioritize public health interventions for diarrheal diseases in areas prone to severe landslides. Such interventions may include improving access to clean water, strengthening sanitation systems and providing rapid response health services during landslide events. Public health campaigns promoting hygiene practices and the use of water purification methods can also mitigate the risk of diarrheal outbreaks. In addition, integrating disaster risk reduction strategies, such as water source protection and early warning systems, could reduce the health burden associated with landslides.

The study revealed significant association between reported drought severity and reported diarrheal disease with individuals exposed to severe droughts reporting higher rates of diarrheal illnesses compared to those less exposed or not exposed. The lack of a significant reported difference between people who were exposed to drought and those not exposed and who became sick with malaria can be linked to the biology of malaria transmission, which depends on the presence of stagnant water for mosquito breeding. Severe drought conditions often lead to reduced water availability, limiting the formation of breeding sites for mosquitoes. Similarly, typhoid fever, while linked to contaminated water, may not show a significant reported association with drought severity in this case, possibly due to localized factors such as water storage practices or existing water access infrastructure. In contrast, the significant increase in reported diarrheal diseases with drought can be explained by the scarcity of safe and reliable water sources during droughts. Such conditions increase the risk of waterborne diseases. This finding is consistent with an increasing number of studies reporting that droughts exacerbate diarrheal illnesses due to poor water quality and hygiene practices coupled with water scarcity during droughts which forces reliance on unimproved water sources, increasing exposure to pathogens [53–57]. The results highlight the crucial need for interventions to address waterborne diseases during droughts. Efforts should focus on improving access to clean and safe water, such as investing in boreholes, rainwater harvesting systems, and water purification technologies. Public health campaigns emphasizing hygiene practices and safe water use can also help mitigate the risks of diarrheal diseases. In addition, governments and aid organizations should strengthen drought preparedness and response strategies by ensuring the availability of emergency water supplies in vulnerable communities. Reported flood severity was found to correlate with reported increased cases of malaria, diarrhea and typhoid fever, with rates being highest among individuals reporting exposure to severe floods, moderate among those reporting moderate flood exposure and lowest among those reporting mild floods exposure. Flooding creates favorable conditions for the proliferation of waterborne and vector-borne diseases. Stagnant water after floods provides breeding grounds for mosquitoes, leading to increased malaria transmission. Other studies found similar findings showing that the changes in temperature and rainfall had consequences of increasing droughts, floods and incidences of pests and diseases, with serious implications on the population health [58, 59] with an increased incidence of malaria [60]. It was observed an increase of malaria cases after severe floods in Kasese in Uganda [20].

These findings present the broader implications of climate variability on public health systems particularly in resource-limited settings, aligning with existing literature, which has consistently highlighted the link between flooding and disease outbreaks. For instance, a global observational study showed increased number of new malaria and typhoid fever cases significantly correlated with longer duration of floods [61]. Furthermore, malaria and diarrheal disease have been linked to floods [62], and yet damaged water and sanitation infrastructure due to floods was found to result into drinking water contamination, water scarcity and increased competition for water, all of which are associated with water-borne diseases like cholera and typhoid fever [63, 64]. Water scarcity, water resource conflicts and contamination have been documented to manifest in the study area [24], and increasing potential for public health challenges. Our findings underscore the urgent need for targeted public health interventions in flood-affected areas. Measures such as improving drainage, distributing mosquito nets, ensuring access to clean drinking water, and strengthening health services during and after floods are critical to reducing disease burden. These findings also reinforce the importance of integrating disaster risk management with public health strategies, as promoted by the World Health Organization [65].The increase of malaria cases is mostly associated with the presence of floods and the increase of temperature that create favourable environmental conditions to mosquito breeding and the development of larva in the flood water [24, 45]. A high frequency of malaria has been seen in regions which were previously free of malaria such as western Uganda due to the variation of climate conditions [23–25]. There is a need to define special mechanisms to control malaria in the region and reinforce prevention measures regarding climate/environmental hazards on one hand but also the preventive measures for malaria on the other hand. It is also important to understand if people have access to mosquito nets in the region and if they are using them properly. Furthermore, studies conducted elsewhere such as Madagascar, India, and other regions of Uganda found similar results showing that lack of WASH facility, people consuming turbid and unsafe water, and the habit of not boiling water expose people to multiple outbreaks [17, 66–69].

The cases of reported typhoid fever were most strongly associated with reports of floods and were equally distributed between people who reported being affected and those who reported not being affected by drought and landslides. This situation needs particular attention for a proper mitigation strategy. Floods, landslides, and drought were all reportedly associated with the exacerbation of typhoid risk which should be the primary focus for interventions. Other studies found that consumption of unreliable water sources such river or unprotected water wells was linked to outbreak including increase of cases of typhoid fever [7, 17, 18, 26, 30, 69].

Researchers have shown the importance of community engagement and involvement while responding to health crises [6, 69–71]. It was found that it is important to involve the community through the whole process of crisis response and make them the frontline actors to expect a smooth response [71]. Organisations need to focus on the risk factors described here and work closely with local communities to map (determine) the real community needs and act on them to relieve suffering for health improvement. These interventions should be co-designed and co-implemented with community members. Studies showed that people who experienced multiple or long-lasting crises develop coping mechanisms that need to be strengthened for a successful response by involving them in the activities planning, implementation, and evaluation [69–73]. Implementers would have a lot to learn from them and focus and/or adapt interventions on community needs.

Causal conclusions cannot be drawn due to limitations in the data. They show the association between reported exposure to climate and / or environmental hazards and the reported occurrence of enteric infectious diseases. Therefore, additional research is needed to confirm causality. It is, important to understand how people—and communities—who were affected by multiple hazards due to climate changes have coped to maintain their normal life. Did they get support from outside? Did they cope with the situation by themselves? What has made their coping mechanisms successful? There is a need to conduct in-depth studies, not only to deeply understand whether and how these vulnerable, affected populations developed resilience mechanisms, but also to determine—and quantify—how this situation impacted their health status, including infectious diseases.

## Conclusion

The study highlights the significant health effects of environmental hazards such as floods, landslides, and droughts on the affected populations in Eastern Uganda. The findings demonstrate that reported malaria and typhoid fever were not significantly associated with the reported severity of droughts or landslides, reported diarrheal diseases showed a correlation with both hazards, with higher frequency observed among individuals who reported being exposed to severe events. Reported floods, in particular, were associated with increased cases of reported malaria, typhoid and diarrheal diseases, emphasizing their wide-reaching health implications. The study underscores the role of water and sanitation systems in mitigating the health effects of environmental hazards. Events such as floods and droughts disrupt access to clean, safe water, forcing communities to rely on unimproved and contaminated sources, thereby increasing vulnerability and the risk of disease outbreaks. There is a need to define and implement strategies to reduce exposure to hazardous events. Such results call for urgent investment in infrastructure, such as improved water, sanitation systems, and drainage systems, and implementation of preventive measures, including water purification, community hygiene education supported by local public health outreach, and disaster preparedness strategies. The study complements global evidence on the health effects of climate extremes and reinforces the importance of integrating public health and disaster risk reduction strategies. Policymakers and other stakeholders must prioritize interventions that enhance community resilience to hazards, particularly in vulnerable regions. Addressing the risk factors of waterborne and vector-borne diseases, improving access to clean water, and strengthening health systems can significantly reduce the disease burden associated with climate / environmental hazards. Future research should focus on long-term monitoring of health outcomes in hazard-prone areas. In addition, there is a need to evaluate the effectiveness of mitigation and adaptation strategies over time to provide a clear understanding of the dynamic relationship between climate/environmental hazards and public health, ultimately informing better policy and practice.

## Data Availability

The datasets used and analysed during the current study are available from the lead and the corresponding authors on reasonable request.
